# Extinction of chromosomes due to specialization is a universal occurrence

**DOI:** 10.1038/s41598-020-58997-2

**Published:** 2020-02-07

**Authors:** Jason Wilson, Joshua M. Staley, Gerald J. Wyckoff

**Affiliations:** 1 University of Missouri-Kansas City School of Medicine, Department of Biomedical and Health Informatics, Kansas City, 64108 Missouri USA; 2 Kansas State University College of Veterinary Medicine, Department of Diagnostic Medicine/Pathobiology, Olathe, 66061 Kansas USA; 3 University of Missouri-Kansas City School of Biological and Chemical Sciences, Department of Molecular Biology and Biochemistry, Kansas City, 64108 Missouri USA

**Keywords:** Genomics, Genome evolution, Genetics, Evolutionary biology

## Abstract

The human X and Y chromosomes evolved from a pair of autosomes approximately 180 million years ago. Despite their shared evolutionary origin, extensive genetic decay has resulted in the human Y chromosome losing 97% of its ancestral genes while gene content and order remain highly conserved on the X chromosome. Five ‘stratification’ events, most likely inversions, reduced the Y chromosome’s ability to recombine with the X chromosome across the majority of its length and subjected its genes to the erosive forces associated with reduced recombination. The remaining functional genes are ubiquitously expressed, functionally coherent, dosage-sensitive genes, or have evolved male-specific functionality. It is unknown, however, whether functional specialization is a degenerative phenomenon unique to sex chromosomes, or if it conveys a potential selective advantage aside from sexual antagonism. We examined the evolution of mammalian orthologs to determine if the selective forces that led to the degeneration of the Y chromosome are unique in the genome. The results of our study suggest these forces are not exclusive to the Y chromosome, and chromosomal degeneration may have occurred throughout our evolutionary history. The reduction of recombination could additionally result in rapid fixation through isolation of specialized functions resulting in a cost-benefit relationship during times of intense selective pressure.

## Introduction

The human Y chromosome has lost its ability to recombine with its once homologous partner, the X chromosome, except in its pseudoautosomal regions (PARs) at the termini of the X and Y chromosomes^1–3^. This has resulted in the majority of the Y chromosome’s gene content being inherited as a unit, known as the human MSY (male-specific region of the Y chromosome)^4^. Suppression of recombination occurred at five discrete time points, probably caused by inversions, that integrated each segment into the MSY and initiated the degradative processes^5^ that resulted in wide-spread gene deletion and loss^1,2,6,7^. These evolutionary strata show a continuum of degeneration that is highly correlated with the age of X-Y gene pairs within each stratum^1,2,4^. The oldest of which contains only four remaining genes, including the sex-determining factor SRY^8^. The degenerative nature of the Y chromosome has led some researchers to suggest it may lose all functional genes and become extinct in as little as 5 million years^8–10^, an evolutionary phenomenon that has been observed in other species^11–13^. Recent research, however, suggests that the Y chromosome has maintained a stable assortment of genes for the last 25 million years^3,14,15^ through effective purifying selection on single-copy genes^16^, and intrachromosomal gene conversion of ampliconic sequences^17–21^. Despite conflicting views on the terminal fate of the Y chromosome, functional specialization and biased gene retention^22–24^ on the Y chromosome is believed to be unique in the genome^25^ and may have played an essential role in Y chromosome degeneration.

The remaining functional genes in the human MSY fall into three classes: X-degenerate, ampliconic, and X-transposed^3,4,26^. The X-transposed sequences are a result of an X-to-Y transposition that occurred after the divergence of the human and chimpanzee lineages, approximately 3-4 million years ago^4,26^. These sequences remain 99% identical to their X counterparts^4^. In contrast, the X-degenerate sequences are single-copy MSY genes that are surviving relics of the ancestral autosomes from which the sex chromosomes evolved^4^. With the notable exception of SRY, these genes are functionally coherent^25^, and ubiquitously expressed^1,14,17^. Their homologous X counterparts also disproportionately escape X-inactivation and are subject to stronger purifying selection than other X-linked genes^17^. Thus, researchers have suggested that this class of sequences is dosage-sensitive and potentially haplolethal^17^. The last class of functional genes in the MSY consists of nine protein-coding gene families that have undergone various levels of amplification^4^. Unlike the ubiquitously expressed X-degenerate genes, the ampliconic gene families are expressed primarily or exclusively in the testes^4,18^ and rely on intrachromosomal gene conversion to offset the degenerative nature of the MSY^18–21^. Surviving Y-linked genes were therefore retained through two evolutionary mechanisms: effective purifying selection on single copy dosage-sensitive genes^16^ and intrachromosomal gene conversion of ampliconic sequences^17–21^.

Wide-spread gene loss accompanied by preferential retention appears to be a unique phenomenon. A review of genomic evolution, however, suggests that these trends are not unique to the Y chromosome, with the relevant literature rarely being cross-cited^27^. Recent research suggests that the ancestral vertebrate karyotype was much larger than previously estimated, consisting of an estimated 54 chromosomes^28^ resulting from two ancestral whole-genome duplication (WGD) events^28–31^. The majority of genes following a WGD event are rapidly lost or pseudogenized due to loss of function mutations^7,32–34^. This loss has also been shown to continue on a power scale^33,35^. Consequently, a large portion of the ancestral vertebrate chromosomes has been subsequently lost through fusion in the descent of the human lineage^28,31^, explaining the apparent haphazard gene content of most autosomes^1^. Highly expressed genes^36^, dosage-sensitive protein complexes^34,37^, and transcriptional and developmental regulators and signal transducers, however, are preferentially retained^30,33,38–40^. Furthermore, these genes have been maintained through purifying selection^37^, a trend that has been observed in ubiquitously expressed genes throughout the genome^41–45^. The factors that led to the biased retention of ubiquitously expressed single-copy genes, therefore, appear not to be restricted to the evolutionary history of the Y chromosome and have been observed in the events following large scale duplications. The biased acquisition of male-advantage traits on the Y chromosome is a subject of more considerable ambiguity in the context of genomic duplications.

Subfunctionalization has been shown to increase the likelihood a gene will be preserved in duplicate due to partial loss of function mutations in both copies^46^. This targeted divergence of the duplicates may lead to differential tissue expression of the paralogs^34,35,45,47–49^ and has been proposed to occur frequently following WGD events^50^. If the remaining functions are under selective constraint, the duplicates will likely remain in the population^47^. A lack of genome-wide representation of subfunctionalized gene pairs, however, suggests that this may be a transition phase to neofunctionalization due to an absence of purifying selection on the redundant portions of the gene^51^, an evolutionary phenomenon known as the subneofunctionalization model^52^. In 2009, Wilson and Makova suggested that suppression of recombination could be thought of as a duplication event and showed X-Y genes followed similar patterns of evolution following recombination suppression as duplicated paralogs^53,54^. Following a review of experimental data, they also concluded that the acquisition of unique expression patterns and functions might have contributed to the retention of Y-linked genes. Strong expression reduction has also been implicated in the evolution of Y genes towards testis specificity^55^. The biased content of male reproductive genes on both sex chromosomes^4,56–58^, therefore, suggests that subfunctionalization of Y-linked genes could explain the initial retention and accelerated divergence of male-advantage genes, as new evolutionary features typically bear marks of their ancestry^50^.

The WGD events at the origin of the vertebrate lineage may have had significant impacts on biological complexity and evolutionary novelties of the time due to the large-scale increase in genetic redundancy^38^. The mechanisms by which this was achieved and the selective pressures resulting in differential chromosome survival remain unknown. If the evolutionary history of the Y chromosome provides a model of genomic evolution, it would suggest that large scale duplication events allowed genes to subfunctionalize and experience periods of relaxed purifying selection through relief from pleiotropic constraints that were operating on single-gene loci^46^. It has also been hypothesized that the Y chromosome’s long-term fragility may be driven by short-term selective pressures^59^, the most obvious of which is the accumulation of sexually antagonistic alleles in a non-recombining portion of the genome^59–65^, a phenomenon that is supported by the transposition of male-advantage genes into the MSY from autosomes^1,3,4,14,66–68^. The rapid evolution of male reproductive genes^45,69–71^ and the implication of inversions in local adaption^7,72^, however, suggest that functional isolation may become selectively favored even in the absence of sexually antagonistic traits under certain circumstances, despite the deleterious effects of reduced recombination. In order to test these hypotheses, and in lieu of the large amount of literature pertaining to expression, we analyzed the nonsynonymous to synonymous mutation rate (${{\rm{K}}}_{{\rm{a}}}\,{\rm{/}}\,{{\rm{K}}}_{{\rm{s}}}$) of 6,734 human genes with surviving mammalian orthologs in the context of their Gene Ontology (GO) annotations and chromosomal locations to determine if functional specialization and genomic isolation convey a selective advantage, respectively.

## Results

### Functional diversity of orthologs

Two primary paths to survival have occurred on the Y chromosome. Broadly expressed dosage-sensitive genes have been maintained through purifying selection^16^, while amplification and gene conversion have supported testis-specific genes^17–21^. Selection for conservation through amplification and gene conversion of testis-specific sequences and the rapid evolution of male reproductive genes^45,69–71^ suggest that adaptability may be a selected phenomenon. Evolution of testis-specific functions is also believed to have preceded amplification on the Y chromosome^17^, suggesting subfunctionalization may have facilitated their initial retention^45^ and subsequent divergence by relieving redundant portions of the genes from adaptive constraint.

**Figure 1:**
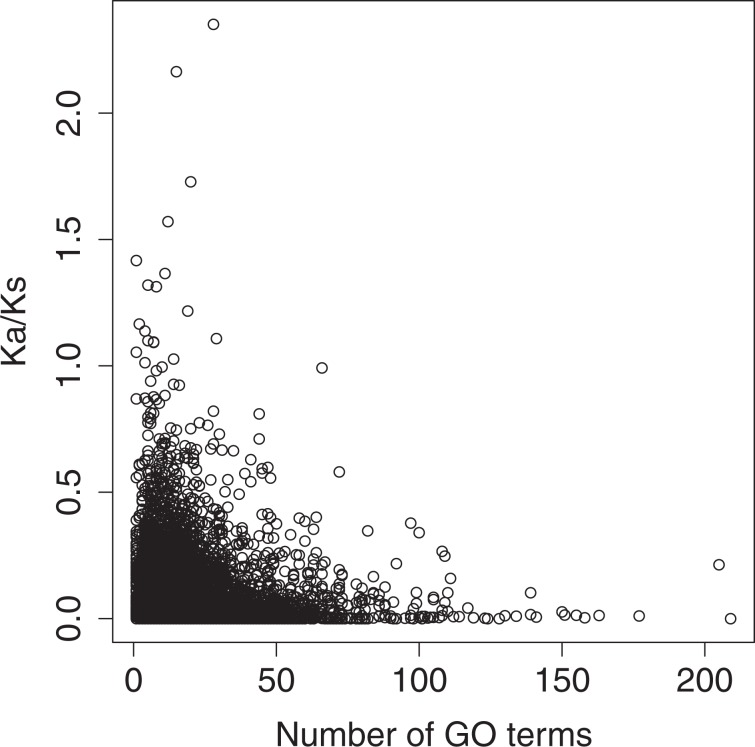
Ortholog ${{\rm{K}}}_{{\rm{a}}}\,{\rm{/}}\,{{\rm{K}}}_{{\rm{s}}}$-GO Relationship: Average ${{\rm{K}}}_{{\rm{a}}}\,{\rm{/}}\,{{\rm{K}}}_{{\rm{s}}}$ values of 6,734 human genes across their related orthologs plotted against their number of GO terms. Each data point represents a human gene with surviving mammalian orthologs (n = 6,734). The x-axis corresponds to the number of gene ontology annotations that were found to be associated with each gene. The y-axis corresponds to a human gene’s average K_a_/K_s_ value across its related species comparisons. The results of a gamma regression analysis indicated the association was statistically significant (p = $8.25\times 1{0}^{-20}$). This figure was generated using R statistical software (Version 3.5.3).

To determine if this is a universal trend, we analyzed the divergence of human genes across their mammalian orthologs to provide a conservative estimate of the degree to which newly duplicated genes may diverge^73^ following subfunctionalization. Summary statistics can be found in Supplementary Table S1. The results of our analysis suggest that a human gene’s average ${{\rm{K}}}_{{\rm{a}}}\,{\rm{/}}\,{{\rm{K}}}_{{\rm{s}}}$ across its related orthologs and number of GO annotations are positively skewed (skew = 3.92 & 3.06, respectively) (Supplementary Fig. S1). Additionally, average ${{\rm{K}}}_{{\rm{a}}}\,{\rm{/}}\,{{\rm{K}}}_{{\rm{s}}}$ is zero-inflated. This suggests that the majority of orthologous genes are under purifying selection and related to a small set of functions. As a gene’s average ${{\rm{K}}}_{{\rm{a}}}\,{\rm{/}}\,{{\rm{K}}}_{{\rm{s}}}$ value appears to be negatively associated with its number of GO annotations (Fig. 1), a gamma-hurdle model was employed (see methods) to determine the statistical significance of this relationship. Our results suggest that a gene’s average ${{\rm{K}}}_{{\rm{a}}}\,{\rm{/}}\,{{\rm{K}}}_{{\rm{s}}}$ decreases with increasing numbers of functional annotations (p = $8.25\times 1{0}^{-20}$) (Supplementary Fig. S2), and the probability that it is entirely conserved increases (p = $5.25\times 1{0}^{-9}$, odds-ratio = 1.011) (Supplementary Fig. S3).

The results of this analysis suggest that genes with high functional diversity are under more intensive purifying selection than their more functionally specific counterparts. These findings parallel those showing higher levels of purifying selection on broadly expressed essential genes throughout the genome^41–45^ as well as on the Y chromosome^17^ and suggest an association between the two. We conclude that functional specificity and reduced expression are associated with relaxed purifying selection, suggesting that subfunctionalization of duplicated paralogs could result in differential tissue expression^34,35,45,47–49^ and accelerated protein divergence.

### Genomic isolation of functional annotations

Next, we were interested in determining if functional isolation can provide a selective advantage in the absence of sexual antagonism. The rapid evolution of male reproductive genes^45,69– 71^ and the implication of inversions in local adaption^7,72^ suggest that the localization of functionally related genes may accelerate protein divergence and facilitate adaptability. To determine if localization of genes related to a given function is associated with reduced purifying selection, we analyzed the average ${{\rm{K}}}_{{\rm{a}}}\,{\rm{/}}\,{{\rm{K}}}_{{\rm{s}}}$ of GO annotation’s related sequence comparisons with respect to the genomic distribution of their related genes. Summary statistics can be found in Supplementary Table S2. GO annotations show positively skewed distributions for their number of associated genes (skew = 34.68), number of chromosome arms they are expressed on (skew = 2.58), and average ${{\rm{K}}}_{{\rm{a}}}\,{\rm{/}}\,{{\rm{K}}}_{{\rm{s}}}$ (skew = 2.85) (Supplementary Fig. S4). This suggests that the majority of functional annotations we analyzed are carried out by a limited number of genes, are expressed in specific locations and under purifying selection. Their relationships with one another, however, suggest all three trends are not typically present at the same time.

**Figure 2:**
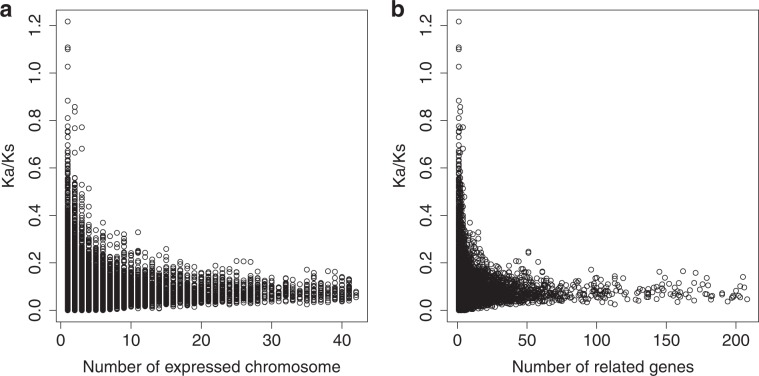
GO ${{\rm{K}}}_{{\rm{a}}}\,{\rm{/}}\,{{\rm{K}}}_{{\rm{s}}}$ Relationships: (**a**) Average ${{\rm{K}}}_{{\rm{a}}}\,{\rm{/}}\,{{\rm{K}}}_{{\rm{s}}}$ values of the 11,016 GO terms with unique gene sets in our dataset across each term’s related genes and human sequence comparisons plotted against the number of chromosome arms a given GO term was found. (**b**) Average ${{\rm{K}}}_{{\rm{a}}}\,{\rm{/}}\,{{\rm{K}}}_{{\rm{s}}}$ values of the same GO terms plotted against their number of related genes (trimmed within 3 SD of the mean number of genes for clarity). Each data point represents a GO annotation (n = 11,016). (**a**) The x-axis corresponds to the number of chromosome arms a given GO annotation was expressed on (the number of chromosome arms containing a gene that was associated with the given GO annotation). The y-axis corresponds to a GO annotation’s average K_a_/K_s_ value across its related genes and their species comparisons. The results of a gamma regression analysis indicated the association was statistically significant (p = $7.01\times 1{0}^{-19}$). (**b**) The x-axis corresponds to the number of genes a given GO annotation was associated with. The y-axis corresponds to a GO annotation’s average K_a_/K_s_ value across its related genes and their species comparisons. The results of a gamma regression analysis indicated the association was not statistically significant (p = 0.05). The accompanying figures were generated using R statistical software (Version 3.5.3).

A GO term’s average ${{\rm{K}}}_{{\rm{a}}}\,{\rm{/}}\,{{\rm{K}}}_{{\rm{s}}}$ value appears to be negatively associated with the number of chromosome arms it is expressed on and its number of related genes (Fig. 2). Due to the positive skewed nature of these distributions (Supplementary Fig. S4), a gamma model with a log link was used to determine if a function’s number of related genes or expressed chromosome arms is significantly associated with its average ${{\rm{K}}}_{{\rm{a}}}\,{\rm{/}}\,{{\rm{K}}}_{{\rm{s}}}$. Increasing the number of genes or expressed chromosome arms related to a given function, however, increases the probability one ${{\rm{K}}}_{{\rm{a}}}\,{\rm{/}}\,{{\rm{K}}}_{{\rm{s}}}$ value is non-zero. Zero average ${{\rm{K}}}_{{\rm{a}}}\,{\rm{/}}\,{{\rm{K}}}_{{\rm{s}}}$ values in the context of this analysis, therefore, are not informative and were removed from the analysis, negating the need for a hurdle method. The two predictive variables were fit separately to determine their individual effects. The results of our analyses suggest a function’s average ${{\rm{K}}}_{{\rm{a}}}\,{\rm{/}}\,{{\rm{K}}}_{{\rm{s}}}$ decreases with the number of chromosome arms it is expressed on (p = $7.01\times 1{0}^{-19}$, Supplementary Fig. S5), however, a function’s number of related genes was non-significant (p = 0.05, Supplementary Fig. S6). This suggests that genomic isolation is more strongly associated with relaxed purifying selection than a function’s number of related genes. The non-significance of a function’s number of related genes additionally suggests that low ${{\rm{K}}}_{{\rm{a}}}\,{\rm{/}}\,{{\rm{K}}}_{{\rm{s}}}$ values for annotations expressed on a large number of chromosome arms cannot be attributed to convergence to the genome-wide average alone.

We expect that the majority of functions related to a large number of genes and expressed throughout the genome are higher-level ontology functions. Genes that are beneficial in increased dosage, however, are preferentially retained following duplication events^73,74^. Thus, large scale duplications may have resulted in the stability of higher-level functions, while relieving more redundant duplicates from adaptive conflict. We conclude that functional isolation is associated with relaxed purifying selection on the genes related to that function, potentially through relief from background selection acting on more highly conserved linked sites^75^. This finding parallels the accelerated evolution of Y-linked genes following recombination suppression and suggests isolation of functions may accelerate sequence divergence of their related genes through relaxation of purifying selection. These findings provide only a modest estimate of the extent to which protein functions may diverge in isolation when recombination is suppressed or following a WGD event when genetic redundancy is at its peak.

### Potential retention of functionally related haplogroups

**Figure 3:**
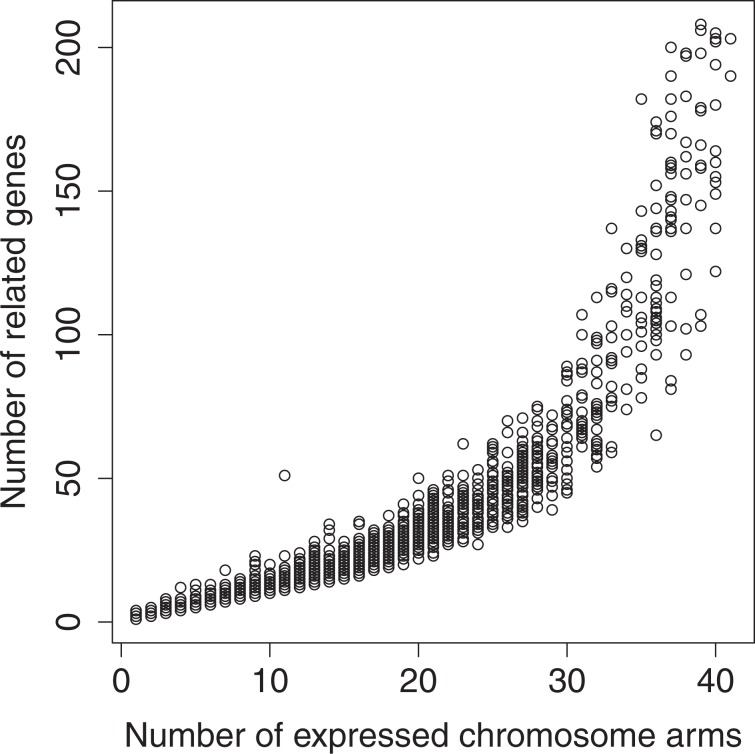
GO Chromosome Arm and Gene Relationship: Number of genes related to each GO term plotted against the number of chromosome arms it was found (trimmed to within 3 SD of the mean number of genes for clarity). Each data point represents a GO annotation (n = 11,020). The x-axis corresponds to the number of chromosome arms a given GO annotation was expressed on (the number of chromosome arms containing a gene that was associated with the given GO annotation). The y-axis corresponds to the number of genes a given GO annotation was associated with. The results of a gamma regression analysis indicated the association was statistically significant (p < 0.0005). This figure was generated using R statistical software (Version 3.5.3).

A GO annotation’s number of associated genes also appears to increase exponentially with the number of chromosome arms it is expressed on (Fig. 3). This relationship was fit using gamma regression and a log link, the results of which were highly significant (p < 0.0005, Supplementary Fig. S7). For a GO annotation’s number of related genes to increase in this manner, the genes on a given chromosome arm must be moderately functionally related. This suggests that the retention of genes following large-scale duplication events may operate at the haplogroup level, a trend that is predicted due to the dosage-sensitive nature of protein complexes. Chromosomes enriched with blocks of functionally related genes that are beneficial in increased dosage would show the highest levels of gene retention. Thus, the functional coherence of the Y chromosome could be attributed to a lower content of functionally related haplogroups that were beneficial in increased dosage on the ancestral autosomes.

### Biased retention of orthologs on existing chromosomes

The ancestral vertebrate chromosomes displayed substantial differences in gene number, potentially as a result of more significant gene deletion and loss on chromosomes with a smaller number of resulting genes^28^. This has led to speculation of systematic biases in the deletion of duplicates on a subset of chromosomes following rediploidization, which may have resulted in the chromosome’s eventual loss^28^. We were interested in determining if this systematic bias could be attributed to the gene content of the pre-duplicated chromosomes from which they were derived. The results of our chromosome analysis show human chromosome arms have normally distributed numbers of orthologous genes (Shapiro-Wilk 0.97, p = 0.34) and average ${{\rm{K}}}_{{\rm{a}}}\,{\rm{/}}\,{{\rm{K}}}_{{\rm{s}}}$ values (Shapiro-Wilk 0.98, p = 0.72) (Supplementary Table S3 and Fig. S8), and that a chromosome arm’s number of genes and GO annotations are linearly related (p = $5.68\times 1{0}^{-40}$, adjusted R^2^ = 0.985, Supplementary Figs. 9 and 10). Density of orthologs on existing chromosome arms, however, was found to be non-normally distributed (Shapiro-Wilk 0.912, p = 0.002). This is due to biased ancestral gene conservation on a subset of chromosomes. Despite differential average ${{\rm{K}}}_{{\rm{a}}}\,{\rm{/}}\,{{\rm{K}}}_{{\rm{s}}}$ rates, selection at the chromosome arm level since the divergence of mammals does not appear to influence the number or density of orthologs on a given chromosome (Supplementary Fig. S11).

In contrast, we found that arms of chromosomes that have retained large clusters of genes resulting from the ancestral WGD events contain a disproportionate number of mammalian orthologs in our analysis. Approximately 35% of genes still exist in duplicate copies^39^, and several paralogs resulting from WGD events^31,32^ (also known as ohnologs) were retained as quadruplicates^28^. These include clusters containing the four HOX regions on chromosomes 2, 7, 12, and 17, as well as the MHC region on chromosome 6 containing ohnologs on chromosomes 1, 9, and 19 that are a result of single pre-duplicated regions^28^. The gene content of chromosomes 14 and 15 have also been shown to be almost entirely derived from individual pre-duplicated chromosomes^28^. The arms of these chromosomes show some of the higher levels of mammalian ortholog retention in our analysis, and chromosomes 17 and 19 have the highest ortholog densities in the genome (Table 1 and Supplementary Fig. S12).

**Table 1:** Chromosome arms summary statistics: Includes number of genes with surviving orthologs, number of GO terms, density of genes or orthologs/Mb, and average ${{\rm{K}}}_{{\rm{a}}}\,{\rm{/}}\,{{\rm{K}}}_{{\rm{s}}}$ of all genes and their species comparisons on a given chromosome arm. Sorted by number of genes.

	Number of Genes	Number of GO Terms	Density	Avg K_a_/K_s_
1p	394	3057	3.19	0.14
1q	354	3096	2.82	0.17
2q	295	2710	1.99	0.14
11q	290	2426	3.55	0.17
17q	289	2630	4.97	0.15
5q	272	2401	2.05	0.14
12q	258	2369	2.64	0.12
15q	231	2130	2.78	0.13
7q	229	2352	2.31	0.16
10q	228	2172	2.43	0.13
3q	218	1950	2.03	0.14
6p	200	1682	3.34	0.16
14q	197	1881	2.19	0.12
3p	197	2035	2.17	0.12
2p	193	2015	2.06	0.14
6q	192	1754	1.73	0.15
4q	188	2099	1.34	0.14
9q	178	1857	1.87	0.15
19q	170	1782	5.24	0.15
8q	168	1685	1.68	0.15
19p	154	1362	5.88	0.14
11p	136	1451	2.55	0.13
Xq	128	1322	1.35	0.13
16p	122	1029	3.32	0.14
13q	121	1156	1.25	0.14
16q	120	1296	2.24	0.13
17p	115	1457	4.58	0.12
7p	114	1213	1.90	0.12
22q	112	1399	3.13	0.13
20q	109	1296	3.00	0.12
12p	99	1026	2.79	0.17
8p	89	1085	1.97	0.15
18q	89	1127	1.44	0.14
Xp	80	894	1.31	0.13
4p	74	878	1.48	0.13
10p	67	790	1.68	0.11
5p	64	787	1.31	0.15
9p	62	865	1.44	0.19
20p	58	838	2.06	0.15
21q	49	542	1.41	0.14
18p	29	455	1.57	0.12
Yq	1	10	0.02	0.09
Yp	1	7	0.10	0.16

**Figure 4:**
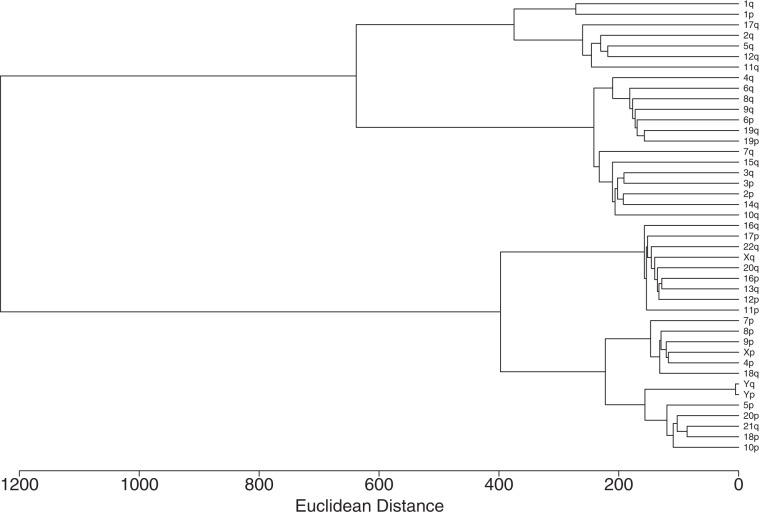
Chromosome Arm Dendrogram: Hierarchical clustering of the chromosome arms based on functional relatedness of their genes. Hierarchical clustering of the chromosome arms based on functional relatedness of their genes. Chromosome arm functionality was obtained by deriving weighted frequencies for each GO annotation within our dataset. Frequencies were weighted based on the specificity of the GO annotation to a given chromosome arm. Distance measure is Euclidean. Multi-node clusters are joined based on minimum increase in within group variance.

To determine the extent to which these chromosomes remain functionally related, aside from their conserved gene families, we performed hierarchical clustering of the chromosome arms based on a weighted frequency (see methods) for each GO annotation on a given arm in our dataset. The results of our dendrogram (Fig. 4) indicate several trends in the functional relationships between genes of chromosome arms. The two top-level clusters appear to be differentiated based on the number of related functions retained on the chromosome arms (Table 1), a result that was expected given clustering with Euclidean distance. We additionally found lower level clustering of chromosome arms that include both the ancestral HOX and MHC regions. These include the clustering of chromosome 2q, 12q, and 17q, as well as 6pq, 19pq, and 9q. This suggests that the ancestral WGD events have had a profound impact on the retention and organization of mammalian orthologs throughout the human genome.

As stated earlier, specific classes of genes are preferentially retained following WGD events. Chromosomes that have maintained a large portion of their ancestral genes are therefore a result of the gene content and functional annotations of the pre-duplicated chromosomes from which they were derived. It has been hypothesized that the specialization of the Y chromosome is a result of the number of functional genes initially present on the ancestral autosomes^57^, a hypothesis supported by the low functional gene density of the X chromosome^2,58^. In our present analysis, we also found low ortholog density on both the X chromosome, and chromosomes that are orthologous to the chicken Z chromosome (chromosomes 5, 9, and 18^58^). However, we did not find as significant of a disparity in ortholog density between the X chromosome and the human genome-wide average (2.33) relative to overall gene density comparisons^58^, suggesting that ancestral gene density may be less heavily influenced by the invasion of interspersed repeats. Biased gene deletion following the ancestral WGD events resulting in low gene density on a subset of chromosomes suggests that several chromosomes were pre-adapted to specialize similar to the sex chromosomes. Furthermore, chromosomal rearrangements would be under less negative selection in these regions.

## Discussion

Since its discovery, the perceived functional importance of the Y chromosome has grown exponentially within the scientific community and now may provide further insight into chromosomal evolution following the ancestral WGD events at the origin of the vertebrate lineage. Our present analysis, in conjunction with existing literature, has shown that evolutionary trends believed to be unique to the Y chromosome are observed in the events following large-scale duplications and are still present in mammalian ortholog comparisons. These include higher levels of purifying selection on functionally diverse, ubiquitously expressed genes^41–45^, as well as reduced purifying selection on genomically isolated protein functions. The biased distribution of ancestral mammalian genes on chromosomes primarily derived from single pre-duplication chromosomes additionally suggests that gene retention was dependent on the gene content of the ancestral chromosome from which they were derived, and this retention may persist over long periods of evolutionary time. The conservation of the functionally coherent, potentially haplolethal X-degenerate sequences through purifying selection^16^, the rapid evolution of ampliconic sequences expressed primarily in the testes^14^, and the pseudogenization and loss of redundant sequences are consistent with a large-scale duplication event. This suggests that the Y chromosome may serve as a model for chromosome evolution following a large-scale duplication event.

Examining suppression of recombination on the Y chromosome in the light of large-scale duplication events has essential implications for karyotypic evolution at the onset of the vertebrate lineage. It has been proposed that the ancestral WGD events contributed to the proliferation of vertebrates during the Cambrian period due to the increase in genetic variation and tolerance to environmental conditions^38,76^. Recent research suggests that all extant vertebrate karyotypes are descendants of an ancestral marine chordate consisting of 17 chromosome pairs that underwent two successive WGDs^28^. Rapid loss through the fusion of seven chromosomes between duplications and the loss of an additional five chromosomes following the second duplication resulted in an ancestral Amniota karyotype of 49 chromosomes with highly differential gene content^28^. The smaller size of extant genomes, therefore, suggests a consistent pattern of karyotype reduction following the ancestral WGD events^28^, and speciation rates have been shown to be strongly correlated with chromosomal evolution rates^77^.

Duplication events should occur at a fitness cost, and an optimal gene copy number should exist^73^. Duplicate genes, therefore, would be subjected to three potential evolutionary fates: retention of genes that are beneficial in increased dosage, inactivation of genes that are harmful in increased dosage, and a period of neutral evolution of redundant sequences. Consequently, the observed gene retention on a given chromosome could be attributed to its density of genes that were beneficial in an increased dosage and subsequently retained through purifying selection. Loss of chromosomes due to widespread gene inactivation of detrimental duplicates, however, should have occurred early and ubiquitously, contributing little to the evolutionary novelties and speciation observed at the time. Considering Y chromosome evolution as a potential model for other chromosomes following a large-scale duplication event and high selective pressure would suggest an alternative hypothesis: chromosomal rearrangements may have protected large regions of the genome from gene flow, which allowed isolated genes to diverge until complete reproductive barriers were formed^78^.

The specialization of SRY as the sex-determining factor appears to have played a significant role in X-Y divergence, as its emergence is correlated with the first stratification event that reduced recombination between the neo-sex chromosomes^79^. Single gene sex determination alone should not select for recombination suppression^80^. However, the presence of gonadal dysgenesis in XY individuals with an SRY deletion^81^ and sterility of XX individuals containing an inactivated copy of SRY^82–87^ suggests that multiple genes are required to produce fertile offspring. The accumulation of sexually antagonistic alleles in a non-recombining portion of the genome could have provided a sufficient selective advantage that outweighed the deleterious effects of reduced recombination due to their synergistic effects on fertility. Despite Mueller’s ratchet being implicated in the early stages of Y chromosome degeneration^88^, genetic decay due to strong positive selection resulting in hitchhiking events is believed to be responsible for its extensive divergence and continued degeneration^88–91^. This is supported by the stepwise repression of recombination^1^ and the correlation of Y-degeneration with levels of female promiscuity in related species comparisons^3,14,15,92^. This suggests that strong positive selection on mutually beneficial alleles at linked sites may drive recombination suppression to become selectively favored.

Ohno’s original model of genetic evolution suggesting that newly duplicated genes would be functionally redundant and able to escape purifying selection^93^ has mixed empirical evidence^54,73^. The events of large-scale duplications, however, create an environment in which newly duplicated genes or complexes that are beneficial in increased dosage may be retained through purifying selection, while the remainder of duplicates would show a continuum of redundancy. The scale of such duplications would allow a small subset of genes to achieve a beneficial mutation. If one mutation resulted in a novel function or further specialized a gene towards one of its respective functions, the likelihood of the gene being retained would increase^46^. Our analysis has also shown that increasing its functional specificity may relax purifying selection, resulting in further divergence. In the event that this new, beneficial mutation occurred on a highly redundant chromosome, the additional reduction of purifying selection due to isolation of a function in an environment with little to no background selection may selectively specialize the chromosome. The survival of the remaining neutrally evolving sequences on that chromosome would depend on their acquisition of functionally related beneficial mutations. If the chromosome bearing this specialized function captured a pair of alleles that together significantly increased the organism’s fitness, selection for recombination suppression may result in an inversion becoming prevalent in the population. As observed on the Y chromosome, the resulting chromosome would now contain a complex of genes maintained through purifying selection, as well as a subset of specialized genes that are rapidly evolving resulting in a period of extensive divergence from its homologous counterpart.

The probability that a new inversion captures an advantageous haplotype can be high^72^; however, for an inversion to become fixed when sexually antagonistic alleles are not present the selective advantage would have to strongly outweigh the negative fitness consequences of reduced recombination^94^. In 1973, Leigh Van Valen showed that the probability of extinction of a population was constant over time and suggested an evolutionary arms race where survival is dependent on a population’s ability to adapt to changing selective pressures^95^. During times of intense selective pressure, selection for rapid fixation of a highly advantageous haplotype may have driven recombination suppression to become selectively favored due to the reduced effective population size and increased fixation rate. Recombination suppression events, such as inversions, in the absence of sexual antagonism, would have markedly different evolutionary consequences. This is due in part to inversions only reducing recombination in heterozygotes^7^. If the inversion is driven to fixation, recombination would resume between the new homologous chromosomes. In isolated populations, this divergence from the ancestral chromosome may have been sufficient to create a reproductive barrier, such as in the divergence of ancestral *Equus* populations^96^. As evidenced by the Y chromosome, recombination suppression can also occur progressively^80^ and may be related to continued selection for newly introduced, functionally related, beneficial alleles. Subsequent inversions resulting from extended periods of intense selective pressure on the associated functions would continue to drive the degeneration of the chromosome through successive hitchhiking events, increasing its long-term fragility.

For the Y chromosome, or any significantly degraded chromosome to go extinct, its functions would need to be replaced elsewhere or no longer under selective constraint to prevent fitness consequences^24^. Relaxation of selection at the locally adapted sites (e.g., predator/prey adaption), however, would render the genes functionally inert, and selection for recombination resumption between ubiquitously expressed genes would result in fusion events becoming selectively favored. Thus, prolonged strong selection for specialization would have driven a subset of ancestral chromosomes to extinction. As this pertains to the fate of the human Y chromosome, continued selection for localization of sexually antagonistic traits, reduction of female promiscuity resulting in less intensive sexual selection, and its recent stability may suggest it is here to stay. Its continued survival in species still experiencing strong sexual selection, however, may be suspect.

It is worth noting that an apparent contradiction in this logic is the ZW sex-determining chromosomes in avian lineages in which females are the heterogametic sex. Similar to the evolution of the Y chromosome, suppression of recombination in the W chromosome has resulted in significant degeneration of its ancestral gene content^97^. Those that remain functionally active have been shown to be ubiquitously expressed and are believed to be essential to both sexes^97^. In contrast, the W chromosome lacks genes coding for female-advantage traits^97^, suggesting that selection for specialization has not resulted in its degeneration. To reduce the recombination between mutually beneficial, sexually antagonistic alleles, however, an inversion can occur in either chromosome^80^. DMRT1 has also been implicated as the sex-determining locus in the ZW system and is present only on the Z chromosome^98,99^, suggesting testes development may function through a dosage-dependent mechanism. In conjunction with a lack of dosage-compensation observed in the ZW system^100^, the degeneration of the W chromosome is still a result of male-driven positive selection, only on the opposite chromosome.

The chicken Z has been found to be orthologous to portions of human chromosomes 5, 9, and 18 while the human X is orthologous to chicken chromosomes 1 and 4^58,97^. Due to a lack of structural similarity with their respective orthologous regions, researchers have suggested the chicken Z and human X chromosome were not predisposed to become sex chromosomes and their low gene density is a result of convergent evolution^58^. Chromosomes 5, 9, and 18, however, show similar levels of ortholog density as the X chromosome in our analysis, as well as, some of the most substantial differences in mammalian ortholog content between the arms of individual chromosomes. This phenomenon is most likely a result of the arms being derived from different ancestral chromosomes that underwent fusion events^28^. Chromosome 9p also contains the ortholog of the believed avian sex-determining locus DMRT1 and is functionally related to the short arm of the X chromosome in our analysis. This may indicate that the convergent evolution of the sex chromosomes is a result of the differential fusion of ancestral chromosomes containing sex-related genes, and the process of sex chromosome evolution further lowered overall gene density to their current state. However, further research needs to be conducted to determine the significance of this relationship.

The results of our analysis in the context of existing literature present a model by which chromosomes, and therefore populations, rapidly evolved at the onset of the vertebrate lineage. The large-scale duplication events allowed a subset of genes to subfunctionalize, thereby reducing pleiotropic constraints and accelerating evolutionary rates. The isolation of these genes on redundant chromosomes further relieved purifying selection, resulting in a period of rapid chromosomal evolution and divergence due to specialization. If this divergence alone did not create a reproductive barrier, the chromosome’s eventual loss due to a change in adaptive pressures would have resulted in differential karyotypes of isolated populations. Thus, the extinction of chromosomes due to specialization is not unique to the Y chromosome, or sex chromosomes in general.

## Methods

In the present analysis, we examined the divergence of human genes from their mammalian orthologs with respect to their GO annotations and chromosomal locations. For mammalian genes, the probability a newly arisen nonsynonymous mutation is fixed, relative to what is expected under neutrality, is resolved by the strength of selection^101^. The nonsynonymous to synonymous mutation rate (${{\rm{K}}}_{{\rm{a}}}\,{\rm{/}}\,{{\rm{K}}}_{{\rm{s}}}$ ratio) is commonly utilized as a measurement of this selective strength, with low values suggesting strong purifying selection and high values indicating relaxed purifying selection and/or positive selection^101^. By definition, human orthologs are identical by descent^102^ with at least one other species in our analysis. The use of mammalian ortholog comparisons in conjunction with GO annotations, therefore, allowed us to analyze how gene protein functions influence the patterns of selection that led to the differential divergence of ancestral mammalian genes.

### Data collection

Divergence data was collected from The Searchable Prototype Experimental Evolutionary Database (SPEED)^103^. SPEED contains orthologous sequence comparisons of nine species including human (*Homo sapiens*), chimp (*Pan troglodytes*), rhesus macaque (*Macaca mulatta*), mouse (*Mus musculus*), rat (*Rattus norvegicus*), dog (*Canis familiaris*), cow (*Bos Taurus*), opossum (*Momodelphis domestica*), and chicken (*Gallus gallus*) as a true outgroup. Methodology on the identification of orthologous groups and calculation of divergence data can be found elsewhere^103^.

Orthologous sequence pairs were queried from SPEED for genetic summary information and their related ${{\rm{K}}}_{{\rm{a}}}\,{\rm{/}}\,{{\rm{K}}}_{{\rm{s}}}$ values. Data cleaning was performed using PySpark (Spark version 2.3.1, Python version 2.7.10). Sequence comparisons containing a ${{\rm{K}}}_{{\rm{s}}}$ value of zero or less due to computational error were removed from the analysis. Any sequence comparison set with unusually low ${{\rm{K}}}_{{\rm{s}}}$ values were removed, as they gave spuriously high ${{\rm{K}}}_{{\rm{a}}}\,{\rm{/}}\,{{\rm{K}}}_{{\rm{s}}}$ values. Where multiple comparisons existed, divergence data inconsistencies were resolved by computing a zero-corrected harmonic mean; therefore, more significant weight was given to conservative estimates^104^, and comparisons containing at least one zero ${{\rm{K}}}_{{\rm{a}}}\,{\rm{/}}\,{{\rm{K}}}_{{\rm{s}}}$ value were assigned a ${{\rm{K}}}_{{\rm{a}}}\,{\rm{/}}{{\rm{K}}}_{{\rm{s}}}$ value of zero. Lastly, sequence comparisons that did not include a human comparison with an associated gene name and chromosomal location were excluded. Our resulting dataset included a total of 68,006 comparisons across 10,849 genes.

Gene ontology information was collected from the European Bioinformatics Institute^105^. The most recent version of human gene ontology annotations (9/19/19) was downloaded and joined to their respective genes. The dataset included 19,395 genes and 18,211 GO terms. The validity of GO terms with IEA (Inferred from Electronic Annotation) evidence codes has been questioned due to their inferential nature^106^. The quality of IEA terms, however, has significantly improved and rival those inferred by curators^107^. To alleviate potentially biased numbers of GO annotations on well-studied genes, IEA terms were also retained. After joining with the ortholog dataset and removing genes that lacked annotation, our final dataset included 6,734 annotated genes across 14,121 GO terms. IEA, IDA (inferred from direct assay), ISS (Inferred from Sequence or structural Similarity), IBA (Inferred from Biological aspect of Ancestor), IMP (Inferred from Mutant Phenotype) and TAS (Traceable Author Statement) evidence codes were the primary methods of annotation in our dataset at 30.2%, 20.96%, 13.2%, 12.5%, 10.3%, and 7.3%, respectively.

### Data preparation

Single value human gene ${{\rm{K}}}_{{\rm{a}}}\,{\rm{/}}\,{{\rm{K}}}_{{\rm{s}}}$ rates were derived by averaging their respective ${{\rm{K}}}_{{\rm{a}}}\,{\rm{/}}\,{{\rm{K}}}_{{\rm{s}}}$ values across all species comparisons present in the dataset. GO annotation ${{\rm{K}}}_{{\rm{a}}}\,{\rm{/}}\,{{\rm{K}}}_{{\rm{s}}}$ values were obtained by averaging the ${{\rm{K}}}_{{\rm{a}}}\,{\rm{/}}\,{{\rm{K}}}_{{\rm{s}}}$ values of all related genes across all species comparisons. Chromosome arm ${{\rm{K}}}_{{\rm{a}}}\,{\rm{/}}\,{{\rm{K}}}_{{\rm{s}}}$ values were calculated by averaging the ${{\rm{K}}}_{{\rm{a}}}\,{\rm{/}}\,{{\rm{K}}}_{{\rm{s}}}$ values of all genes present on the respective chromosome arm across all species comparisons. Ortholog density was calculated by dividing the number of orthologs present on a given chromosome arm by arm size in Mb. Lastly, the chromosome arms and their related GO annotations were cross-tabulated to obtain the number of times a given function occurs on each arm. Due to their hierarchical nature, GO terms can be broad^106^. This issue was addressed in a context dependent manner for each analysis. Prior to clustering the chromosome arms based on functional relatedness of their genes, the number of times each GO annotation occurred on each chromosome arm was weighted using an algorithm adapted from Martinez and Reyes-Valdés^108^. We considered the average frequency of the ${{\rm{i}}}^{{\rm{th}}}$ GO term among j chromosome arms as,

1 $${\rho }_{i}=\frac{1}{t}\mathop{\sum }\limits_{j=1}^{t}{\rho }_{ij}$$

and defined GO term specificity as the information that its expression provides about the identity of the chromosome arm as

2 $${S}_{i}=\frac{1}{t}(\mathop{\sum }\limits_{j=1}^{t}\frac{{\rho }_{ij}}{{\rho }_{i}}{{\rm{\log }}}_{2}\frac{{\rho }_{ij}}{{\rho }_{i}})$$

${{\rm{S}}}_{{\rm{i}}}$ will give zero if the GO term is expressed on all chromosome arms and max ${{\rm{\log }}}_{2}$(t) if the function is exclusively expressed on a single chromosome arm. We then assigned a weighted frequency for each GO term on each chromosome arm as the product of the GO term specificity and its frequency on a given chromosome arm.

3 $${\delta }_{ij}={\rho }_{ij}{S}_{i}$$

Thus, a higher degree of functional similarity would be found between chromosome arms if their shared functions were absent elsewhere in the genome. This method was also applied to the relationship between genes and their related GO terms. Weighted GO term counts were derived for each gene by summing the specificities of their related GO terms in equation (3). However, the weighted GO counts did not alter the distributions or significances of our ortholog regression analyses. Therefore, raw counts were used for ease of interpretability.

A primary goal of our GO annotation regression analyses was to determine if genomic representation influences the selective pressures exerted on a function. Therefore, all ontology terms were retained in this analysis in order to examine our hypothesis that large scale duplication events may relieve pleiotropic constraints in a subset of genes through increased dosage of essential functions. However, where multiple GO terms contained the same set of related genes, only one term was retained to remove redundant data points. 11,020 terms of the original 14,121 were found to have unique sets of related genes. 11,016 of these were found to have non-zero ${{\rm{K}}}_{{\rm{a}}}\,{\rm{/}}\,{{\rm{K}}}_{{\rm{s}}}$ values and were used in ${{\rm{K}}}_{{\rm{a}}}\,{\rm{/}}\,{{\rm{K}}}_{{\rm{s}}}$ regression analyses.

### Statistical analysis

All regression and distribution analyses were performed in Python (see version above) using the statsmodels API. Due to the strong positive skew of several variables in our dataset, generalized linear models (GLM) were used where appropriate for hypotheis testing. Fitting positively skewed continuous data with a gamma distribution has been shown to perform comparably or outperform lognormal transformations without the need for manual manipulation of the variables^109^ and provides a more flexible model when assumptions of ordinary least squares are violated. A log link was used to maintain a non-linear fit with interpretable coefficients while respecting the domain of the gamma function. The exponential of the coefficient for the intercept and predictor variable, therefore, represent the initial predicted outcome value and rate of change for a one-unit increase in the predictor variable, respectively. This methodology was applied to the relationship between an ortholog’s number of GO terms and its ${{\rm{K}}}_{{\rm{a}}}\,{\rm{/}}\,{{\rm{K}}}_{{\rm{s}}}$ ratio, a GO term’s number of expressed chromosome arms and its ${{\rm{K}}}_{{\rm{a}}}\,{\rm{/}}\,{{\rm{K}}}_{{\rm{s}}}$ ratio, a GO term’s number of related genes and its ${{\rm{K}}}_{{\rm{a}}}\,{\rm{/}}\,{{\rm{K}}}_{{\rm{s}}}$ ratio, and a GO term’s number of expressed chromosome arms and number of related genes. Where statistically meaningful zero values were present, a hurdle method was employed to counteract the calculation error introduced. This entails fitting a gamma distribution to all non-zero data, as well as a binomial distribution to the full dataset to determine the influence of the predictor variable on the probability that the dependent variable is zero^110^. This was applied to the relationship between an orhtolog’s ${{\rm{K}}}_{{\rm{a}}}\,{\rm{/}}\,{{\rm{K}}}_{{\rm{s}}}$ ratio and number of GO terms. The linear relationship of chromosome arm’s number of related genes and GO annotations was fit with ordinary least squares regression without an intercept, as it was nonsensical in the given context. Normality of distributions was determined using the Shapiro-Wilk test which tests the null hypothesis that the data are normally distributed.

Hierarchical clustering of the chromosome arms based on GO annotation content was performed using the cluster package in R (Version 3.5.3). GO term counts were not scaled before distance calculation due to the homogenous nature of the variables. The distance was calculated using Euclidean distance. The linkage measure was determined by obtaining the agglomerative coefficient (amount of clustering structure found) for single, complete, average linkage and Ward’s method using the agnes() function. For our dataset, Ward’s method resulted in the highest agglomerative coefficient and was subsequently used in our clustering analysis. Therefore, multi-node clusters were joined based on minimum increase in within-group variance.

## Supplementary information

Supplementary Information.

## Data Availability

Requests for compiled data materials pertaining to this manuscript should be addressed to J.W. Inquiries pertaining to the use of raw data from the SPEED database should be submitted to J.M.S. email address jstaley2@ksu.edu.

## Code availability

Requests for code should be addressed to J.W.
