# Supplementary material for: Extinction of chromosomes due to specialization is a universal occurrence
Source: Sci Rep. 2020 Feb 7;10:2170. doi: 10.1038/s41598-020-58997-2 (PMC7005762; doi:10.1038/s41598-020-58997-2)
Supplement: Supplementary file 1 — Supplementary Information. [file 41598_2020_58997_MOESM1_ESM.pdf]

Supplementary Table S1: **Gene Summary Statistics**

Statistic	Number of GO Terms	K_a/K_s
mean	18.87	0.1039
stdev	16.61	0.1423
min	1	0
max	209	2.35

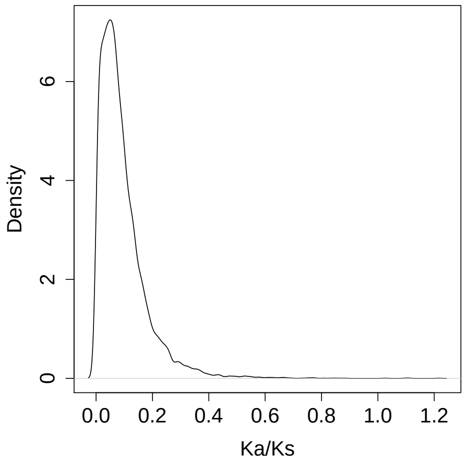

(a)

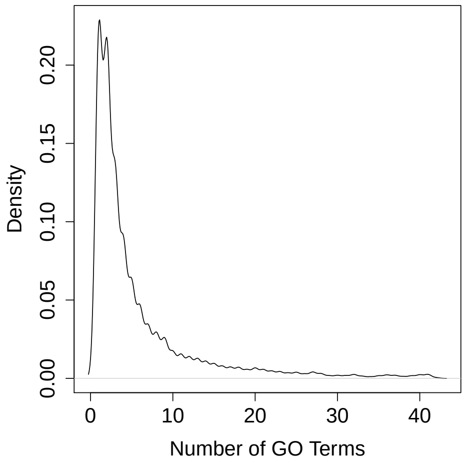

(b)

Supplementary Figure S1: Density plots showing (a) the distribution of average K_a/K_s values and (b) number of related Gene Ontology (GO) terms for the 6,734 human genes with orthologous sequence comparisons in our dataset. For summary statistics see table above. The distribution of average K_a/K_s and number of GO terms both show strong positive skew (skew statistic = 3.92 and 3.06, respectively). The distribution of average K_a/K_s values is also zero-inflated, containing 663 genes that were found to have entirely conserved protein sequences.

**Dep. Variable:**	K_a/K_s	**No. Observations:**	6071
**Model:**	GLM	**Df Residuals:**	6069
**Model Family:**	Gamma	**Df Model:**	1
**Link Function:**	log	**Scale:**	1.6022
**Method:**	IRLS	**Log-Likelihood:**	7037.9
		**Deviance:**	8649.3
		**Pearson chi2:**	9.72e+03

	**coef**	**std err**	**z**	**P>|z|**	**[0.025**	**0.975]**
**Intercept**	-2.0032	0.025	-81.311	0.000	-2.052	-1.955
**Number of GO Terms**	-0.0091	0.001	-9.110	0.000	-0.011	-0.007

Supplementary Figure S2: Generalized linear model (GLM) Gamma Regression Results. Using gamma regression and a log link, non-zero average K_a/K_s values of human genes across their surviving orthologs were fit using an intercept and their number of associated GO terms as predictor variables. The exponential of the coefficient for the intercept and number of GO terms, therefore, represent the initial predicted K_a/K_s value and rate of change for a one-unit increase in number of GO terms, respectively. The intercept, as well as a gene's number of associated GO terms were found to be significant.

**Dep. Variable:**	Zero K_a/K_s	**No. Observations:**	6734
**Model:**	GLM	**Df Residuals:**	6732
**Model Family:**	Binomial	**Df Model:**	1
**Link Function:**	logit	**Scale:**	1.0000
**Method:**	IRLS	**Log-Likelihood:**	-2151.0
		**Deviance:**	4302.0
		**Pearson chi2:**	6.71e+03

	**coef**	**std err**	**z**	**P>|z|**	**[0.025**	**0.975]**
**Intercept**	-2.4511	0.060	-41.024	0.000	-2.568	-2.334
**Number of GO Terms**	0.0117	0.002	5.839	0.000	0.008	0.016

Supplementary Figure S3: GLM Binomial Regression Results. Using binomial regression, the probability a human gene's average K_a/K_s value is zero was fit using an intercept and a gene's number of associated GO terms as the predictor variables. The intercept and a gene's number of related GO terms were both found to be significant. The odds-ratio of a gene being entirely conserved to not can, therefore, be determined by the exponential of the linear equation of predictors.

Supplementary Table S2: Ontology Summary Statistics

Statistic	Number of Related Genes	Number of Expressed Chromosome arms	K_a/K_s
mean	11.216	5.71	0.093
stdev	66.019	7.07	0.087
min	1	1	0
max	4105	42	1.217

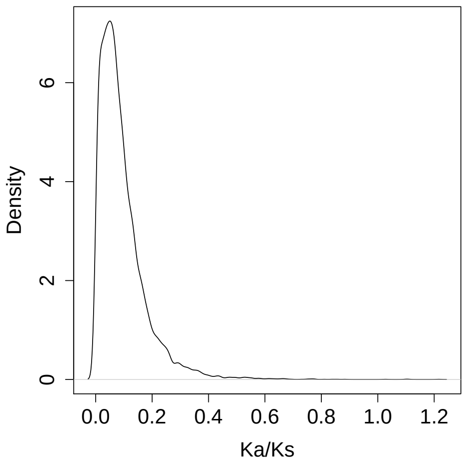

(a)

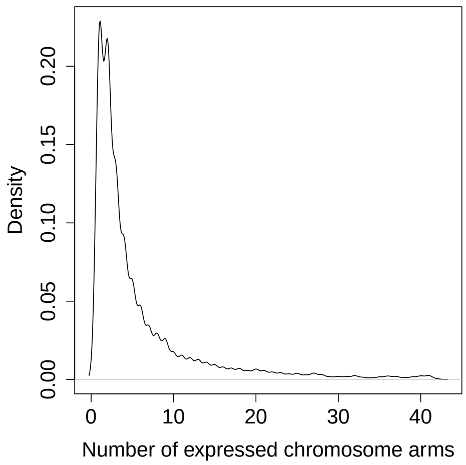

(b)

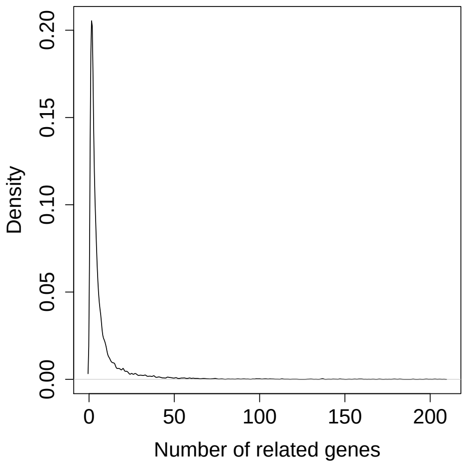

(c)

Supplementary Figure S4: Density plots of a GO annotation's: (a) Average K_a/K_s values, (b) number of expressed chromosome arms, (c) and number of related genes for the 11,016 GO terms with unique gene sets in our dataset. For summary statistics, see table above. GO annotations show positively skewed distributions for their number of related genes (skew = 34.68), number of chromosome arms they are expressed on (skew = 2.58), and average K_a/K_s value (2.85).

**Dep. Variable:**	K_a/K_s	**No. Observations:**	11016
**Model:**	GLM	**Df Residuals:**	11014
**Model Family:**	Gamma	**Df Model:**	1
**Link Function:**	log	**Scale:**	0.81965
**Method:**	IRLS	**Log-Likelihood:**	15374.
		**Deviance:**	9445.7
		**Pearson chi2:**	9.03e+03

	**coef**	**std err**	**z**	**P>|z|**	**[0.025**	**0.975]**
**Intercept**	-2.3145	0.011	-208.731	0.000	-2.336	-2.293
**Number of Expressed Chromosome Arms**	-0.0108	0.001	-8.875	0.000	-0.013	-0.008

Supplementary Figure S5: GLM Gamma Regression Results. Using gamma regression and a log link, non-zero K_a/K_s values for GO annotations averaged across all related human genes and their surviving orthologs were fit using an intercept and the number of chromosome arms they are expressed on. The exponential of the coefficient for the intercept and number of expressed chromosome arms, therefore, represent the initial predicted K_a/K_s value and rate of change for a one-unit increase in chromosome arms expressed, respectively. The intercept, as well as the number of chromosome arms a GO term is expressed on were found to be significant.

**Dep. Variable:**	K_a/K_s	**No. Observations:**	11016
**Model:**	GLM	**Df Residuals:**	11014
**Model Family:**	Gamma	**Df Model:**	1
**Link Function:**	log	**Scale:**	0.87176
**Method:**	IRLS	**Log-Likelihood:**	15298.
		**Deviance:**	9508.0
		**Pearson chi2:**	9.60e+03

	**coef**	**std err**	**z**	**P>|z|**	**[0.025**	**0.975]**
**Intercept**	-2.3706	0.009	-262.717	0.000	-2.388	-2.353
**Number of Related Genes**	-0.0003	0.000	-1.964	0.050	-0.001	-5.57e-07

Supplementary Figure S6: GLM Gamma Regression Results. Using gamma regression and a log link, non-zero K_a/K_s values of GO annotations averaged across all related human genes and their surviving orthologs were fit using an intercept and the number of genes related to a given GO term. The exponential of the coefficient for the intercept and number of related genes, therefore, represent the initial predicted K_a/K_s value and rate of change for a one-unit increase in number or related genes, respectively. The intercept was found to be significant. However, a GO term's number of related genes did not significantly influence its average K_a/K_s value.

**Dep. Variable:**	Number of Genes	**No. Observations:**	11020
**Model:**	GLM	**Df Residuals:**	11018
**Model Family:**	Gamma	**Df Model:**	1
**Link Function:**	log	**Scale:**	0.17410
**Method:**	IRLS	**Log-Likelihood:**	-21580.
		**Deviance:**	2125.0
		**Pearson chi2:**	1.92e+03

	**coef**	**std err**	**z**	**P>|z|**	**[0.025**	**0.975]**
**Intercept**	0.5542	0.005	108.464	0.000	0.544	0.564
**Number of Expressed Chromosome Arms**	0.1554	0.001	276.393	0.000	0.154	0.157

Supplementary Figure S7: GLM Gamma Regression Results. Using gamma regression and a log link, GO term's number of related genes were fit using an intercept and the number of chromosome arms they were expressed on. The exponential of the coefficient for the intercept and number of related genes, therefore, represent the initial predicted number of genes and rate of change for a one-unit increase in number of expressed chromosome arms, respectively. The intercept, as well as the number of chromosome arms a GO term is expressed on were found to be significant.

Supplementary Table S3: Chromosome Arm Summary Statistics

Statistic	Number of Genes	Number of GO Terms	Density	K_a/K_s
mean	156.6	1543.44	2.33	0.1397
stdev	91.56	746.434	1.20	0.0175
min	1	7	0.02	0.0935
max	394	3096	5.88	0.1867

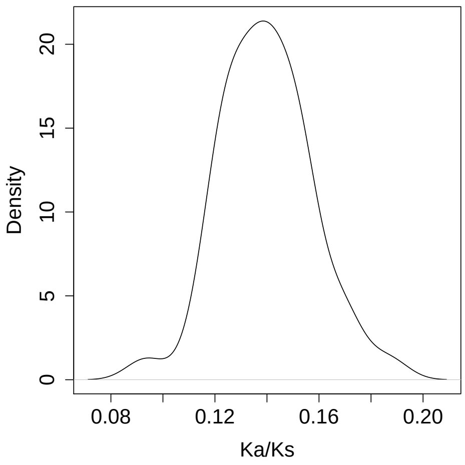

(a)

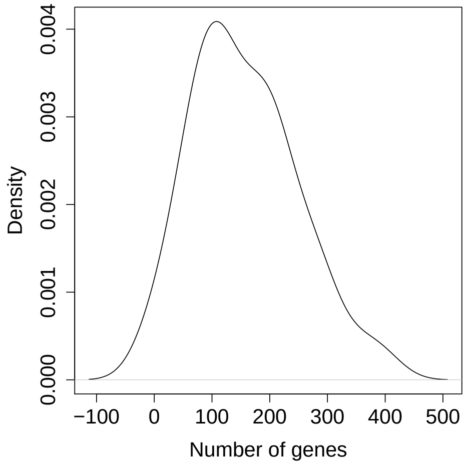

(b)

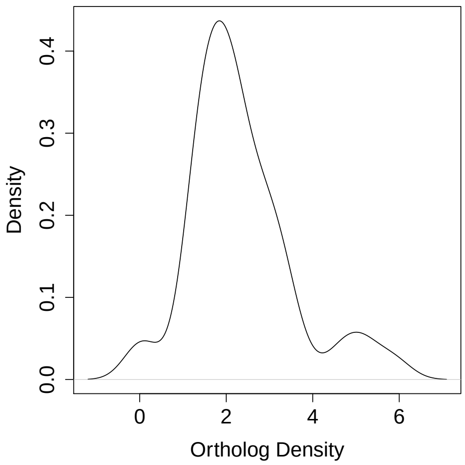

(c)

Supplementary Figure S8: Density plots showing the distribution of (a) average K_a/K_s values, (b) number of orthologous genes, (c) and orthologs/Mb on the 43 chromosome arms. For summary statistics, see table above. The human chromosome arms have normally distributed numbers of orthologous genes (Shapiro-Wilk 0.97, p = 0.34) and average K_a/K_s values (Shapiro-Wilk 0.98, p = 0.72). Density of orthologs, however, is not normally distributed (Shapiro-Wilk 0.91, p = 0.002).

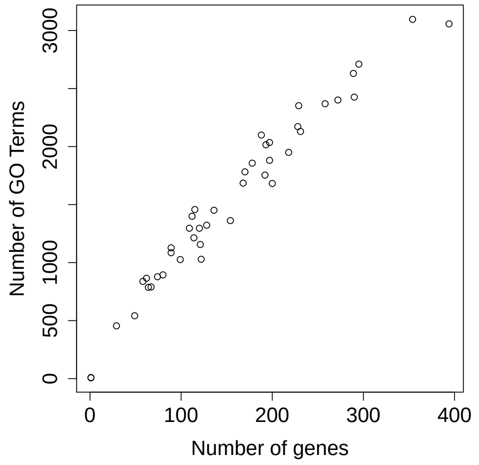

Supplementary Figure S9: A chromosome arm's number of associated GO terms plotted against its number of orthologous genes within our dataset. The relationship was fit with ordinary least squares linear regression, the results of which were significant and statistics can be found below.

**Dep. Variable:**	Number of GO Terms	**R-squared:**	0.985
**Model:**	OLS	**Adj. R-squared:**	0.985
**Method:**	Least Squares	**F-statistic:**	2769.
**No. Observations:**	43	**Prob (F-statistic):**	5.68e-40
**Df Residuals:**	42	**Log-Likelihood:**	-290.76
**Df Model:**	1	**AIC:**	583.5
		**BIC:**	585.3

	**coef**	**std err**	**t**	**P>|t|**	**[0.025**	**0.975]**
**Number of Genes**	9.3872	0.178	52.617	0.000	9.027	9.747

**Omnibus:**	15.170	**Durbin-Watson:**	1.313
**Prob(Omnibus):**	0.001	**Jarque-Bera (JB):**	18.511
**Skew:**	-1.163	**Prob(JB):**	9.56e-05
**Kurtosis:**	5.218	**Cond. No.**	1.00

Supplementary Figure S10: Ordinary Least Squares (OLS) Regression Results. Using linear regression, a chromosome arm's number of related GO terms was predicted based on the number of orthologous genes found on the chromosome arm, the results of which were found to be highly significant ($p = 5.68 \times 10^{-40}$). A chromosome arm's number of functional annotations, therefore, is linearly related to the number of genes on a given chromosome arm by a factor of 9.3872. Our adjusted R-squared value also indicates that this trend should hold across multiple datasets.

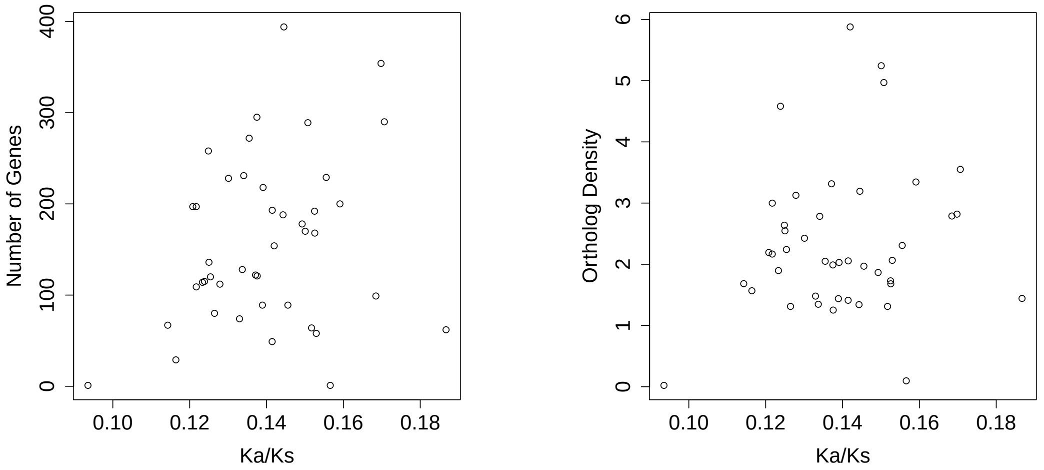

(a) (b)

Supplementary Figure S11: Scatter plots of chromosome arm's (a) number of orthologous genes and (b) orthologs/Mb versus average K_a/K_s values. As stated in the main text, we were unable to find a significant relationship between a chromosome arm's number of related genes or orthologs/Mbp with its average K_a/K_s value. The random distribution of these variables among average K_a/K_s values, therefore, suggests selection at the chromosome arm level since the divergence of mammals has not significantly impacted gene retention.

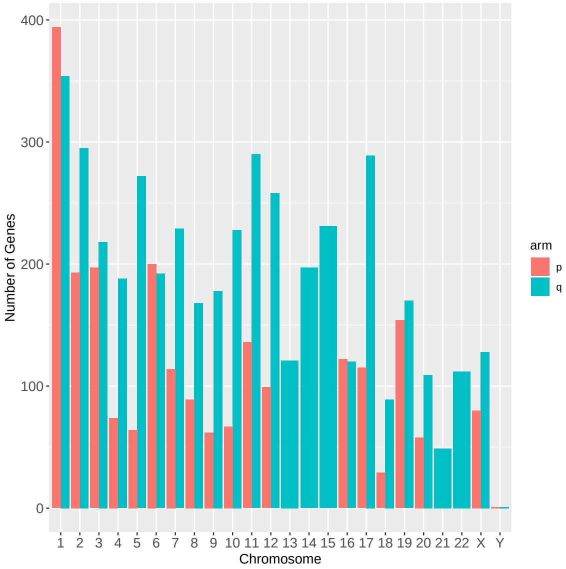

(a)

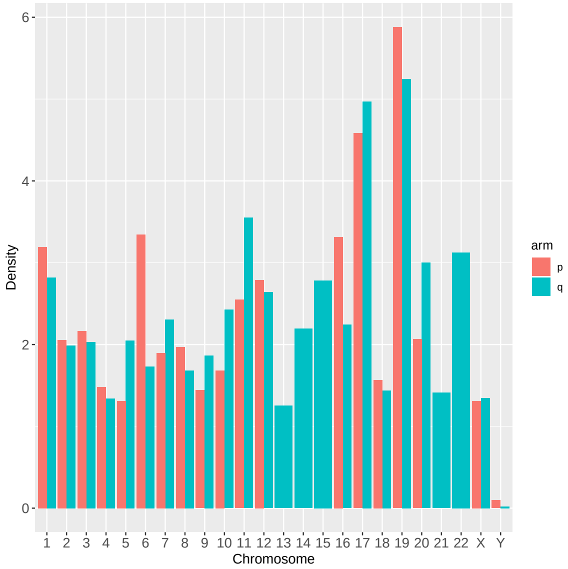

(b)

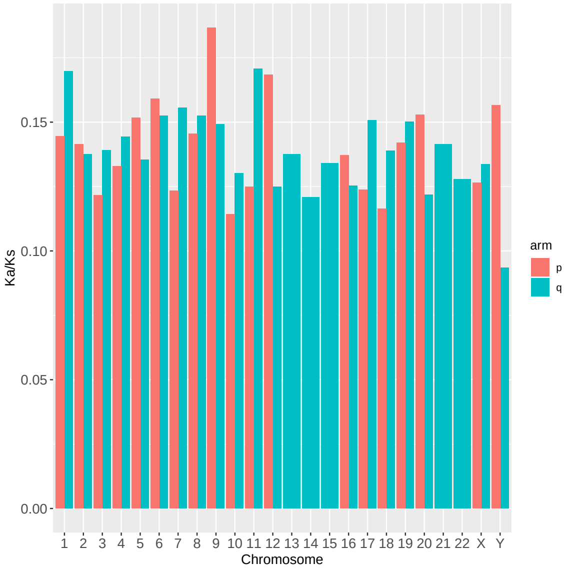

(c)

Supplementary Figure S12: Bar graphs showing each chromosome arm's (a) number of orthologous genes, (b) orthologs/Mb, and (c) average K_a/K_s. The bar graphs provide visual representation of the disparity between the arms of different chromosomes, as well as the separate arms of individual chromosomes. In contrast, we find that the average K_a/K_s values of the chromosome arms do not vary with the same magnitude
